# Developmental origin of a major difference in sensory patterning between zebrafish and bluefin tuna

**DOI:** 10.1111/j.1525-142X.2012.00529.x

**Published:** 2012-03-30

**Authors:** Alain Ghysen, Christine Dambly-Chaudière, Denis Coves, Fernando de la Gandara, Aurelio Ortega

**Affiliations:** a INSERM U710, Laboratoire des Mécanismes Moléculaires des Démences NeurodégénérativesMontpellier, France; b Université Montpellier 2Montpellier, France; c Centre Hospitalier Arnaud de Villeneuve, Laboratoire de Biologie Cellulaire et HormonaleMontpellier, France; d CNRS UMR 5235, Laboratoire de Dynamique des Interactions Membranaires Normales et PathologiquesMontpellier, France; e IFREMER, Laboratoire Aquacole du Languedoc RoussillonPalavas, France; f Instituto Español de Oceanografia (IEO), Centro Oceanografico de MurciaPuerto de Mazarron, Spain

## Abstract

The posterior lateral line system (PLL) of teleost fish comprises a number of mechanosensory organs arranged in defined patterns on the body surface. Embryonic patterns are largely conserved among teleosts, yet adult patterns are highly diverse. Although changes in pattern modify the perceptual abilities of the system, their developmental origin remains unknown. Here we compare the processes that underlie the formation of the juvenile PLL pattern in *Thunnus thynnus*, the bluefin tuna, to the processes that were elucidated in *Danio rerio*, the zebrafish. In both cases, the embryonic PLL comprises five neuromasts regularly spaced along the horizontal myoseptum, but the juvenile PLL comprises four roughly parallel anteroposterior lines in zebrafish, whereas it is a simple dorsally arched line in tuna fish. We examined whether this difference involves evolutionary novelties, and show that the same mechanisms mediate the transition from embryonic to juvenile patterns in both species. We conclude that the marked difference in juveniles depends on a single change (dorsal vs. ventral migration of neuromasts) in the first days of larval life.

## INTRODUCTION

The patterning of sense organs has a major influence on their behavioral function, yet in no case have changes in sensory pattern been satisfactorily explained–-except for the drastic change corresponding to eye loss in the blind cavefish, *Astyanax* (Jeffery, 2008, 2009). A case in point is the lateral line, a system that relies on the activity of mechanosensory hair cells similar to those in the inner ear, and analyzes local water flows. This system is involved in many aspects of fish behavior, from prey detection and predator avoidance to exploration and schooling (Coombs and Montgomery, 1999). The posterior lateral line system (PLL) comprises a large number of superficial sense organs (neuromasts) arranged in defined patterns on the body and tail.

Early development of the PLL involves the long-distance migration of an embryonic primordium, prim1, as originally discovered in amphibians (Stone, 1937), Harrison, 1904 and later confirmed in zebrafish (Metcalfe, 1985) and in tuna fish (Ghysen et al. 2010). During this migration, prim1 deposits five clusters of cells (proneuromasts L1–L5) along the horizontal myoseptum, and a continuous stripe of interneuromast cells (Grant et al. 2005, Lopez-Schier and Hudspeth, 2005). The embryonic PLL is fully differentiated at 2 dpf (days post-fertilization, see Material and Methods for conventions on larval age). The molecular bases of this development have been extensively studied in zebrafish over the past 10 years (reviewed in Ghysen and Dambly-Chaudière, 2007, Friedl and Gilmour, 2009, Ma and Raible, 2009, Aman and Piotrowski, 2010).

**Figure 1:**
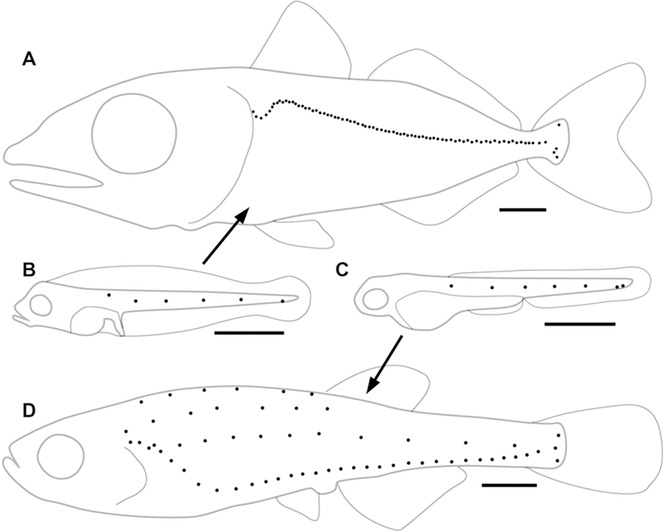
Posterior lateral line (PLL) patterns. *Thunnus thynnus* PLL at juvenile (A) and embryonic (B) stages. *Danio rerio* PLL at embryonic (C) and juvenile stages (D). Black dots represent neuromasts, scale bars: 1 mm.

Embryonic patterns very similar to that formed in zebrafish have been observed in other teleost species (Pichon and Ghysen, 2004). For example, nearly identical patterns are found in late embryos of *Danio rerio* (zebrafish) and *Thunnus thynnus* (bluefin tuna), two highly derived species that belong respectively to the Ostariophysi and Acanthopterygii superorders of teleosts. In both species the embryonic PLL comprises five neuromasts regularly spaced along the horizontal myoseptum, linked by a stripe of interneuromast cells (Fig. 1, Ghysen et al. 2010).

Adult patterns differ widely among fish species, however (Webb, 1989). In the case of zebrafish and tuna fish, juveniles present respectively four parallel lines extending from head to tail in zebra, and a single arched line in tuna (Fig. 1). Here we examine how this difference comes about. We conclude that the mechanisms that mediate the transition from embryonic to juvenile patterns are retained from zebra to tuna, and that the marked difference in juvenile patterns depends on a single difference in late embryonic development.

## MATERIAL AND METHODS

### Spawning and handling of bluefin tuna larvae

Larvae were obtained from spontaneous spawning in cages managed by the Company Caladeros del Mediterraneo S. L. at El Gorguel (Cartagena, Spain) in the frame of the European SELFDOTT project. Spawning began on June 16, 2010, 6 days past new moon (spawning in tuna tends to begin around new moon, and data from the Amami marine station reveal that spontaneous spawning began on new moon on three occasions since 2003, K. Kato, personal communication). Eggs were transferred from El Gorguel to the Ifremer facilities at Palavas (France) prior to hatching and reared for up to 3 weeks. Larger samples were fixed in Spain, kept in fixative and sent later to Montpellier. All labeling and observations were done at University Montpellier 2.

### Larval age

For early larvae, we converted the ages used for bluefin tuna from the usual “days post-hatch” to “days post-fertilization”, to facilitate comparison with zebrafish development. Hatch day corresponds to 2 dpf. For later larvae, huge differences in size are consistently observed within a single batch, and therefore we used the length of the larva as measure of developmental stage. In our hands, the fastest larvae reached 1 cm at 24 dpf. This timing may be due to imperfect rearing conditions as others have reported faster growth, with 9 mm being reached at about 15 dpf (Kawamura et al. 2003).

### Labeling

Larvae were simultaneously labeled for actin by phalloidin labeling, and for acetylated tubulin (present in all neurons and hair cells) by immunolabeling. Briefly, larvae were fixed in cold 4% formaldehyde in PBS for 4–5 h, rinsed 4× 15 min in PBTr (PBS with 0.7% Triton X-100), blocked with 10% goat serum in PBS, incubated overnight at 4°C with anti-acetylated tubulin (monoclonal, Sigma T6793) 1:1000 in PBB (PBS with 1% goat serum, 1% bovin serum albumin), rinsed 3× 15 min in PBTr, incubated 8–10 h in Cy3-coupled secondary antibody 1:4 OO (Jackson Immunoresearch), Alexa Fluor 488-coupled phalloidin (Invitrogen) 1:60 in PBB, rinsed 2× 15 min, left overnight in PBTr, and examined. From 12 dpf on, there was an increased probability of incomplete tubulin labeling, most likely due to penetration problems; this problem was not alleviated by treating the larvae with trypsin or acetone.

### Imaging

All images were taken on a Leica SPE confocal microscope with water-immersion objectives ×20, ×40, and ×63. Because the surface of the fish is not flat, with bulges corresponding to individual somites, and because the primordia, proneuromasts, and interneuromast cells are tightly squeezed between muscles and periderm, we could rarely use maximum projection representation of the stacks as those were uniformly green due to intense muscle actin labeling. Thus all images were pieced from consecutive frames of the stacks. For some figures, pieces from as many as 17 consecutive frames had to be used to illustrate the complete extent of a given feature. In order to minimize interference with the original data, adjacent pieces of the final figure are derived from adjacent frames of the confocal stack in all cases, and the same intensity adjustment was used for all frames of a stack.

## RESULTS

### Embryonic-juvenile transition in zebrafish

**Figure 2:**
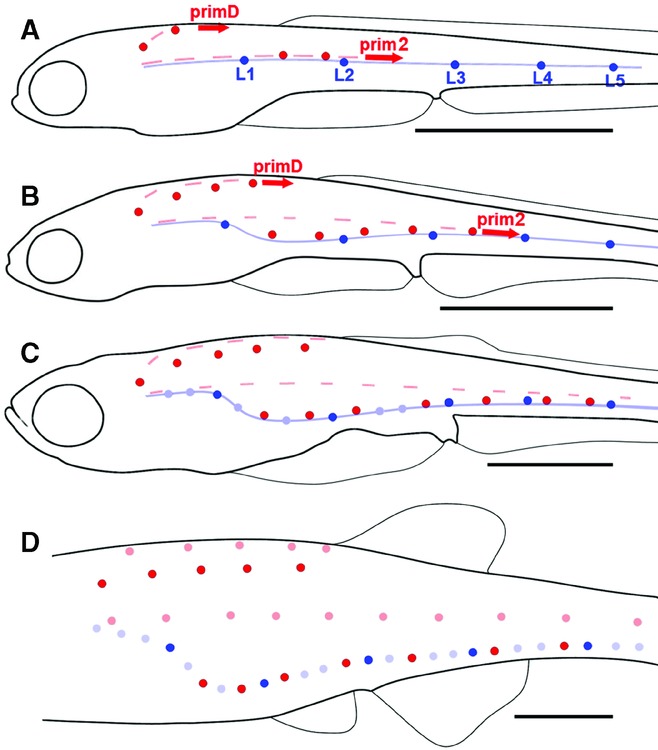
Transition from embryonic to juvenile pattern in zebrafish larvae. (A) The embryonic pattern (blue) is complete at 2 dpf. It comprises five lateral neuromasts (L1–L5) as well as a stripe of interneuromast cells (light blue). (A, B) Migration of post-embryonic primordia primD and prim2, and deposition of their neuromasts (red), extends from 3 to 12 dpf (3–5 mm). In addition to neuromasts, primD and prim2 deposit a discontinuous line of interneuromast cells (pink dashes). (B, C) Ventral migration of neuromasts, and of the stripe of prim1-derived interneuromast cells still attached to neuromasts L1–L5, extends from 4 to 14 dpf (4–6 mm). prim2- and primD-derived interneuromast cells keep their original positions. (C, D) Formation of intercalary neuromasts by local proliferation of prim1-derived interneuromast cells (light blue) extends from 10 to 20 dpf (4.5–7 mm). (D) Formation of intercalary neuromasts by prim2- and primD-derived interneuromast cells (light red) takes place between 15 and 25 dpf (6.5–8 mm). In D, the stripe of prim1-derived interneuromast cells has been omitted for clarity. Scale bars: 1 mm.

The juvenile PLL is a single arched line in tuna fish (Fig. 1A), whereas it comprises four lines in zebrafish (Fig. 1D). The transition from embryonic to juvenile pattern has been elucidated recently in zebrafish (Nuñez et al. 2009). Three processes are involved, which unfold progressively from anterior to posterior, and overlap in time (Fig. 2).

First, a secondary primordium appears near the end of embryogenesis (around 40 hours post-fertilization [hpf]). Part of it, prim2, migrates along the horizontal myoseptum as did the embryonic primordium, and deposits 8–10 secondary neuromasts in between the embryonic neuromasts (red dots, Fig. 2A–C). The other part, primD, migrates dorsally and deposits five to six neuromasts along the dorsal midline until it meets the dorsal fin (Fig. 2A–C). Both prim2 and primD also deposit a discontinuous trail of interneuromast cells (pink dashes, Fig. 2A–C). All neuromasts derived from primD and prim2 have their hair cells polarized along the dorsoventral axis, contrary to the embryonic neuromasts, which are anteroposteriorly polarized (Lopez-Schier et al. 2004).

Second, both lateral and dorsal neuromasts move ventrally (Fig. 2B–D). This movement involves a translocation of the specialized cells that form the pore of the neuromast, through the superficial peridermal cells (Sapède et al. 2002). Due to this displacement, the two lines that were originally lateral and dorsal become respectively ventral and dorsolateral (Fig. 2D). The continuous line of prim1-derived interneuromast cells moves ventrally together with the neuromasts (Fig. B, C).

Third, the interneuromast cells deposited by prim1 proliferate locally and form intercalary neuromasts at all somitic boundaries that are not already occupied by prim1 or prim2 neuromasts (light blue circles, Fig. 2C, D). Interneuromast cells deposited by prim2 and primD likewise form additional neuromasts (pink dots, Fig. 2D). Because the latter do not migrate ventrally and keep their original positions, they form two new lines, lateral and dorsal respectively, thus completing the juvenile pattern.

The juvenile pattern is further amplified during adult life, as every neuromast of the juvenile pattern buds off additional neuromasts that remain closely apposed to form a “stitch” (Ledent, 2002).

### prim2 and primD in bluefin tuna

**Figure 3:**
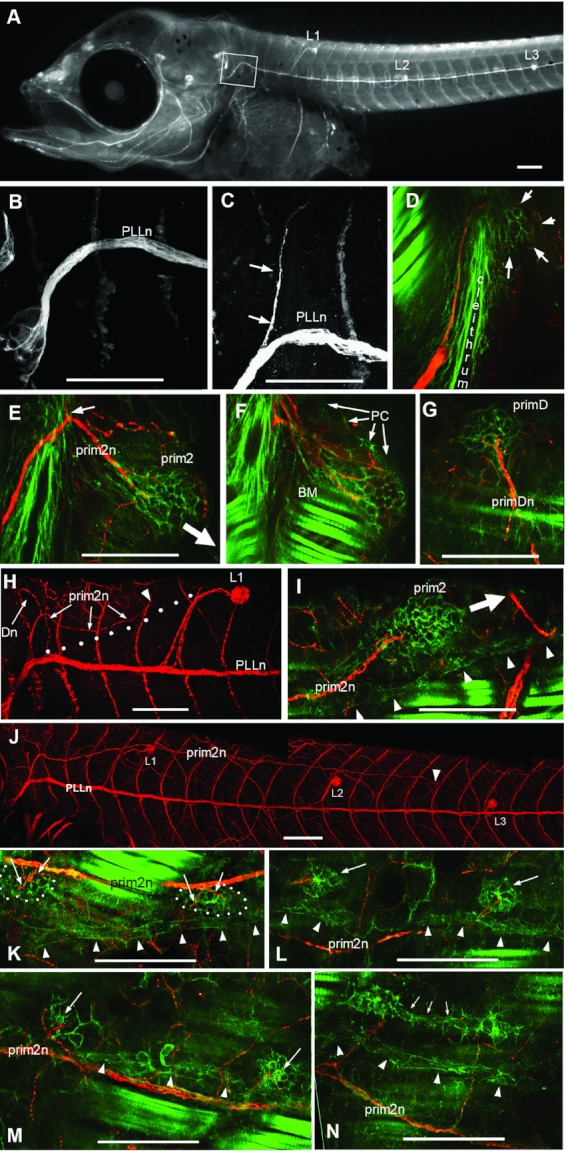
Post-embryonic development of *Thunnus* posterior lateral line (PLL). (A–C) Appearance of a dorsal branch of the PLL nerve (PLLn). (A, B) At 4 days post hatch (corresponding to 6 dpf, 3.6 mm larva) no branch emerges from the PLL; on the next day (4.1 mm), a branch has formed (arrows in C). (D) This branch is lead by a migrating group of mesenchymal cells, prim2 (arrows). (E) The leading axons of the prim2 nerve (prim2n) ramify within prim2. (F) A single confocal frame of the stack used to mount panel E, illustrating the close apposition of peridermal cells (PC), PLL structures, and body muscles (BM). (G) A dorsal branch, primDn, leaves prim2n (arrow in Fig. 2E) and ramifies within a second primordium, primD. (H) prim2n veers away from the horizontal myoseptum (arrows) along a course that corresponds to the stripe of interneuromast cells (dots). The position of prim2 is shown by the arrowhead. (I) At higher magnification, prim2 is seen to migrate just dorsal to the stripe of interneuromast cells (arrowheads). (J) Position of prim2 (arrowhead) at 10dpf (5.2 mm), indicating a rate of migration of about three somites/day. (K) Innervation of prim2-derived proneuromasts by branchlets of prim2n (arrows). (L, M) Proneuromasts assume a rosette-like structure and eventually form pores (arrows in M). (N) prim2-derived neuromasts are (rarely) deposited in close succession, and may even be connected (arrows). prim2-derived neuromasts, and prim1-derived interneuromast cells, keep moving dorsally during larval growth, and progressively separate from prim2n (N). Scale bars: 100 μ.

Embryonic development of the PLL in *Thunnus* involves migration of prim1 and deposition of five neuromasts, L1–L5, interconnected by a continuous line of interneuromast cells, much like in *Danio* (Ghysen et al. 2010). L1 moves dorsally at 3 dpf, followed later by L2 and to a lesser extent by L3 (Fig. 3A). Interneuromast cells are dragged dorsally by the movement of L1–L3. No other change takes place over the next several days.

The first sign of a developing secondary line appears 7 dpf, much later than the onset of prim2/primD migration at 2 dpf in zebrafish (Sapède et al. 2002). A branch arises dorsally from the PLL nerve (Fig. 3A–C), led by a small group of cells, prim2 (Fig. 3D, arrows). At this age the earliest bone of the pectoral girdle, the cleithrum, has expanded dorsally, thus forming a barrier to cell migration (Fig. 3D). prim2 moves around the cleithrum and back toward the horizontal midline as soon as it passes the dorsal edge of the cleithrum (Fig. 3D, E), still followed by axons forming the prim2 nerve (prim2n).

A second nerve branch splits from prim2n as it passes the cleithrum (Fig. 3E, arrow) and is also led by a migrating primordium, primD (Fig. 3G). primD keeps migrating dorsally, and then turns posteriorly along the dorsal midline until it reaches the anterior edge of the dorsal fin, much as it does in zebrafish. Because the dorsal fin is located much more anteriorly in tuna than in zebrafish, however, the dorsal line is much abbreviated in the former. Both prim2 and primD are squeezed between muscles and peridermal cells (Fig. 3F), and can only be resolved by confocal microscopy (see Material and Methods for imaging methods).

The ventral course of prim2 toward the horizontal myoseptum, after it passes the cleithrum (Fig. E, arrow), is soon deflected dorsally again (Fig. 3H*,* the position of the primordium is indicated by the arrowhead). We wondered whether this change in course might result from an interaction of prim2 with the interneuromast cells deposited by prim1, and dragged dorsally by the migration of neuromast L1 (Fig. 3H, the white dots indicate the position of the stripe of interneuromast cells, Ghysen et al. 2010). Confocal analysis reveals that indeed prim2 migrates along the interneuromast stripe, always remaining just dorsal to it (Fig. 3I, the white arrow shows the direction of prim2 migration). As the dorsal migration of embryonic neuromasts is most marked for L1, the path of prim2 progressively converges with the horizontal myoseptum after passing L1 (Fig. 3J).

### Deposition of secondary neuromasts

Much as prim1 did, prim2 deposits in its wake clusters of cells which remain distinct from, and dorsal to, prim1-derived interneuromast cells (Fig. 3K–N, arrowheads). Clusters tend to be elongated soon after deposition (Fig. 3K, dotted outline) but become more rounded soon thereafter. These putative neuromasts, or proneuromasts, are richly innervated by branchlets of prim2n (Fig. 3K, arrows). A few such clusters are deposited anterior to L1, and many more are deposited posterior to L1. The migration of prim2 is much slower than that of prim1, as is also the case in zebrafish. It has reached L1 at 5 mm, L2 at 6 mm, L3 at 7 mm, and L5 at 9 mm (around 22 dpf in our hands, but this timing may be due to imperfect raising conditions as others have reported faster growth, with 9 mm being reached at about 15 dpf, Kawamura et al. 2003).

During this long period, the clusters acquire a well organized rosette-like structure (Fig. 3L, arrows) but do not undergo full differentiation, as no hair cells are formed. Even as late as 20 dpf when prim2-derived proneuromasts have developed pores that would allow the extension of mechanosensory hairs into the surrounding water (Fig. 3M, arrows), still no hair cells are present.

The frequency of prim2-derived neuromasts is about one per somite, but occasionally two neuromasts develop in close vicinity, and can even be linked by intervening cells (Fig. 3N, arrows), although in general they are isolated from each other. During its short journey along the dorsal midline before reaching the anterior edge of the dorsal fin, primD deposits three to four neuromasts.

### Hair cell differentiation

**Figure 4:**
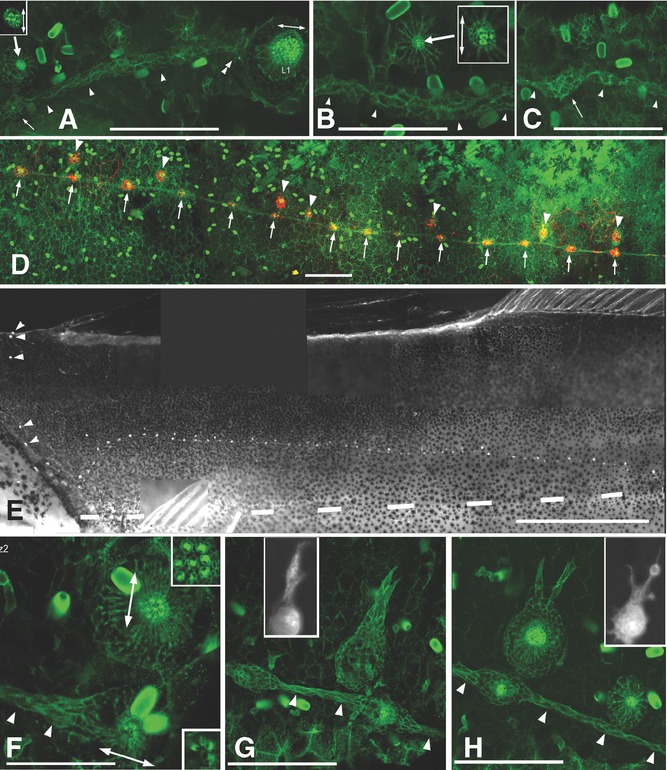
Post-embryonic hair cell differentiation in *Thunnus*. (A, B) In a 24 dpf larva (9 mm), phalloidin-labeled apical hair tufts characteristic of differentiated hair cells simultaneously differentiate all along the body, as illustrated here in prim2-derived neuromasts anterior to L1 (A), and between L3 and L4 (B) in the same larva. Most neuromasts comprise two to four hair cells irrespective of their anteroposterior position, and hair cells are invariably polarized along the dorso-ventral axis (insets). Note in A the continuity of embryonic neuromast L1 and interneuromast cells (double arrowhead). Thin arrows in A, C show incipient intercalary neuromasts. (D) Intercalary neuromasts (arrows) are in line with the stripe of interneuromast cells, whereas prim2-derived neuromasts (arrowheads) are dorsal to it. (E) 1.5 cm larva showing the juvenile pattern of a single arched line, dorsal to the horizontal myoseptum (dashed line), and the diminutive dorsal line (arrowheads). (F) Orthogonal polarization of prim2-derived and of intercalary neuromasts. (G, H) prim2-derived neuromasts extend dorsal processes similar to those observed in zebrafish (black and white insets) when neuromasts form stitches. Scale bars: 1 mm in panel E, 100 μ in all other panels, insets in A, B magnified twice.

As noted previously (Ghysen et al. 2010), not much seems to happen to the PLL over the first 2 or 3 weeks of larval life, and the pattern of differentiated neuromasts remains unchanged from the late embryonic pattern established at 2 dpf. Hair cells begin to differentiate within the secondary neuromasts rather synchronously along the entire length of the larva, at about 1 cm (around 22 dpf). (Fig. 4A, anterior to L1, and B, same larva between L3 and L4). They are all polarized along the dorsoventral axis, orthogonal to the polarization of the embryonic neuromasts.

At this time the line of interneuromast cells has thickened considerably, and local swellings indicate the onset of intercalary neuromast formation along the entire body (thin arrows in Figs. 4A and C). Swellings also appear rather synchronously along the entire PLL (Fig. 4A and C, respectively anterior to L1, and posterior to L3, in the same larva).

At 1.5 cm, a large number of intercalary neuromasts have formed all along the fish. Intercalary neuromasts retain their continuity with the still detectable stripe of interneuromast cells, whereas secondary neuromasts retain their dorsal position relative to this stripe (Fig. 4D).

The axis of hair cell polarization is anteroposterior in prim1-derived intercalary neuromasts, and dorsoventral in prim2-derived neuromasts (Fig. 4F), as is also the case in zebrafish (Lopez-Schier et al. 2004, Nuñez et al. 2009).

At 2 cm, the pattern has become identical to the juvenile pattern, with a seemingly single arched line—not taking into account the diminutive dorsal line (Fig. 4E, arrowheads), which had never been noted previously. At this time, secondary neuromasts form budding structures (Fig. 4G, H) that closely resemble those involved in stitch formation in zebrafish (insets), thus closing the larval-juvenile transition and marking the onset of adult development.

### Ectopic line

**Figure 5:**
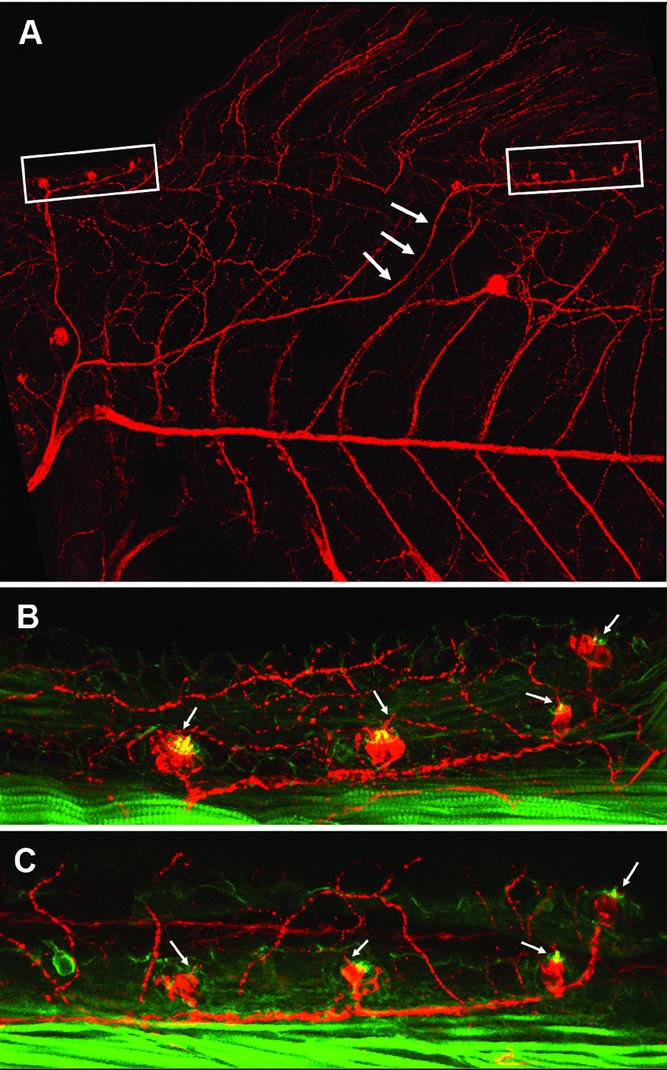
Early differentiation of an ectopic line in a 13 dpf larva. As in normal larvae of this age, primD has formed four to five neuromasts anterior to the dorsal fin (B, boxed in panel A). prim2 has veered dorsally (arrows) instead of following the interneuromast cells past L1, and prim2-derived neuromasts have differentiated precociously (C, boxed in panel A). Arrows point to hair cells kinocilia (red) and stereocilia (green), two clear signs of hair cell differentiation.

We observed one case of abnormal PLL development which throws some light on the difference between primD and prim2 neuromasts. In this case, prim2 failed to migrate back to the horizontal myoseptum along prim1-derived interneuromast cells, and moved dorsally instead (Fig. 5A, arrows). We observed that upon reaching the dorsal midline prim2 migrates posteriorly and deposits clusters of cells in its wake, much like primD does (Fig. 5B, dorsal line, and C, ectopic secondary line). Interestingly, these clusters are fully differentiated at 13 dpf, much earlier than the cluster deposited by prim2 along its normal course. This suggests that the delay in neuromast differentiation is not an intrinsic property of prim2, but is rather a response to local environment. One obvious possibility is that this delay is imposed by the prim1-derived interneuromast cells, which remain closely apposed to the secondary cell clusters.

## DISCUSSION

*Danio* and *Thunnus* belong respectively to the Ostariophysi and Acanthopterygii superorders of teleost fish, which may have diverged as long ago as 290 millions of year (Steinke et al., 2006, but see also Hurley et al. 2007, for remaining uncertainties in the time estimates of major actinopterygian radiations). Accordingly, there is a considerable distance between the two species, even more so because *Thunnus* belongs to the perciforms, a highly derived group within the Acanthopterygians. Among the many derived features of *Thunus* are endothermy, and the presence of a completely independent second dorsal fin, anterior to the ancestral dorsal fin. This “new” fin, a major innovation among teleost fishes, presumably results from an event of duplication-divergence of an ancestral “dorsal fin module” as discussed in Mabee et al. (2002).

**Figure 6:**
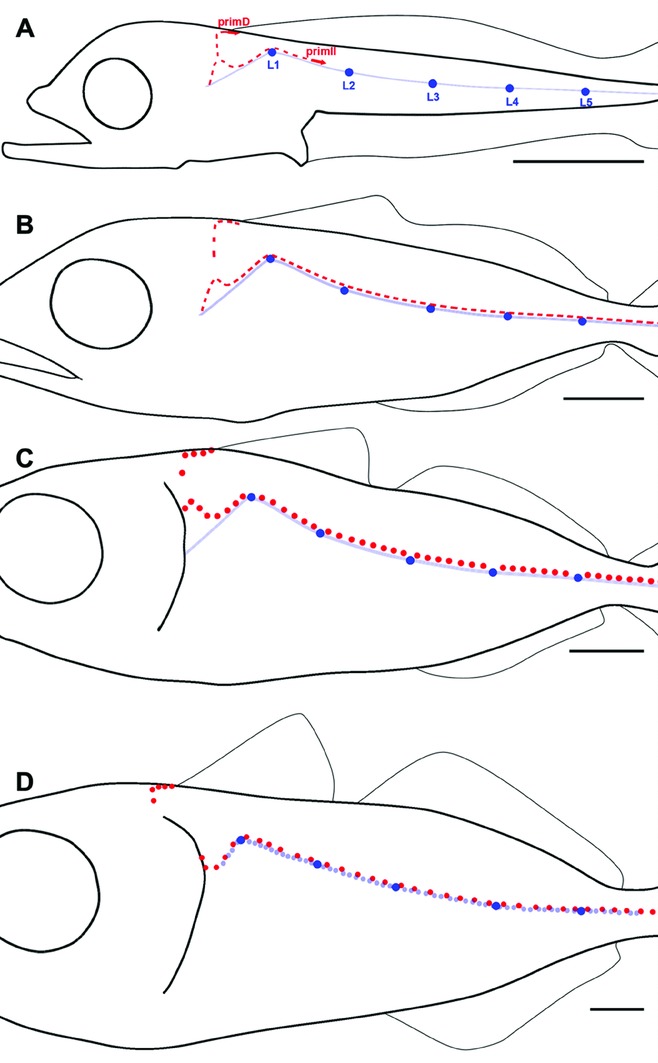
Major steps in posterior lateral line larval development in *Thunnus*. (A) migration paths of prim2 and primD in a 5-mm larva (9 dpf). Both primordia leave small clusters of cells in their wake (red dashes). prim1-derived neuromasts and interneuromasts cells are dark and light blue, respectively. (B) Completion of the prim2 line in a 9-mm larva (20 dpf). (C) Synchronous formation of hair cells in neuromasts of the prim2 line (red dots) in a 10-mm larva (22 dpf). (D) synchronous formation of hair cells in prim1-derived intercalary neuromasts cells (light blue) in a 15-mm larva (25 dpf). Scale bars: 1 mm.

Juvenile PLL patterns also differ widely between *Danio,* where several parallel lines are spread over the trunk and tail, and *Thunnus* with its single, arched line (also observed in many other perciform species). Here we examined to what extent this difference involves the emergence of new developmental processes in either species. The transition between embryonic and juvenile patterns in *T. thynnus* is summarized in Fig. 6, to be compared with the process in *D. rerio*, Fig. 2.

Our results show that the single perciform line actually results from the close apposition of a primary line derived from the embryonic primordium, prim1, and of a secondary line derived from the larval primordium prim2. Thus all mechanisms involved in PLL development appear to be fully conserved between these widely separated teleost species, whose last common ancestor dates from at least 146 million years ago (Hurley et al. 2007). Specifically, the amplification of the embryonic, prim1-derived system through the formation of intercalary neuromasts by interneuromast cells, and the formation of a secondary system set up by prim2 and primD, are fully conserved between the two species. Likewise, the anteroposterior polarization of hair cells in all prim1-derived neuromasts (embryonic or intercalary), and the dorsoventral polarity in all prim2-derived neuromasts, is conserved.

We conclude that the difference between the tuna and zebrafish patterns can be traced back to the fact that the embryonic neuromasts L1–L3 move dorsally in tuna fish, whereas L2–L4 move ventrally in zebrafish. Thus a single, relatively minor change that takes place over the first few days after hatching markedly affects a pattern that lasts the entire life of the fish.

What could be the effect of this change in pattern on the way PLL information is used? There is unfortunately very little information about PLL-dependent behaviors in zebrafish late larvae and juveniles, and none whatsoever for tuna. The remaining part of the discussion will therefore necessarily be speculative, but it provides a plausible account of several puzzling features of PLL development in tuna, not least the inordinate delay in differentiation of secondary and intercalary neuromasts.

One major function of the early line formed by prim1 in zebrafish is its role in detecting predator's strikes (Mc Henry et al. 2009). The progressive development of the juvenile pattern during larval development presumably allows for a higher sensitivity in predator avoidance, as well as for new ways of using PLL information. Given that the dorsal line is derived from primD, its neuromasts are sensitive to dorsoventral water movement, making them well suited to detect surface ripples such as produced by wriggling insects. Conversely the ventral line, with a major complement of prim1-derived, anteroposteriorly polarized neuromasts, may be more sensitive to riverbed reflections. Thus zebrafish may take advantage of the orthogonal polarization of primary and secondary neuromasts, and allocate different functions to the two subsets.

Besides its putative importance in triggering an escape reaction, the embryonic line may also be of special importance in *Thunnus* for prey detection, as the eyes are not at all well developed at hatch day in this species (L. Besseau, personal communication). Early tuna larvae are likely to depend much on their embryonic PLL to detect prey and initiate chasing behavior. The visual system progressively takes over and appears to be the major prey-localizing system at 7 dpf, based on observation of larval behavior in experimental tanks (unpublished observations). Thus the importance of the PLL for prey catching would decline in tuna from paramount at 3 dpf to minimal at 7 dpf, consistent with the observation that no new neuromasts are added between 3 dpf, when the embryonic line is completed, and 20 dpf, when the juvenile pattern begins to form with the differentiation of the earliest secondary neuromasts.

The presence of a second set of primordia, and the orthogonal polarization of prim1- and prim2-derived neuromasts, are retained in bluefin tuna. It is obvious, however, that the detection of surface insects is of no concern in this species, nor is the detection and analysis of ocean bottom. We propose that the later function of the PLL in this species may be essentially social. Tuna fish are known to have an extensive social life, from their habit of hunting in parabolic packs to catch a maximal amount of food, to their tendency to mate during moonless nights. A longitudinal line combining neuromasts with anteroposterior and dorsoventral sensitivities would provide juveniles and adults with a highly sensitive system for perceiving their mate's movements. Further, the existence of a single line may facilitate the establishment of an accurate somatotopic map, the rudiments of which have been demonstrated in zebrafish. (Alexandre and Ghysen 1999, Pujol-Marti et al. 2010, Sato et al. 2010)

The transition from prey detection in the embryonic line to social function in the adult line should pose serious problems of changes in connectivity. The very long pause in tuna fish PLL development between 3 and 20 dpf may facilitate the transition from a simple predator-avoiding, prey-catching behavior relying essentially on the second-order PLL projection to the premotor nucleus of the Medial Lateral Fasciculus (MLFn), as observed in zebrafish (Fame et al. 2006) and tuna fish (AG, unpublished observations), to a more elaborate connectivity. Interestingly, our unpublished observations show that social interactions among tuna are first observed when larvae reach about 1 cm, that is, at the time the juvenile PLL begins to differentiate.
